# Billroth’s Clinic in 1882

**Published:** 1883-09

**Authors:** 


### Billroth's Clinic in 1882.

The *Med. Times and Gaz.*, April 14, 1883, says that Dr. Wolfler states (*Allg. Med. Woch.*, January 23,) that the statistical data of 1882 furnish the interesting fact that the percentage of mortality in Prof. Billroth's clinic presented an enormous difference to that of 1881 ; for while in the former year it was only 6.3, in 1881 it was 9.5 ; and he attributes this difference, in a great extent, to the far more frequent use of iodoform. Among the other unjustifiable objections which have been taken to the employment of this valuable medicinal agent is its liability to induce erysipelas; but how erroneous this statement is, is shown by the fact that there are under its use far fewer wound-diseases than formerly. In the year 1881 there were 822 patients treated in the clinic, of which number 82 (9.5) died ; while of the 722 patients treated in 1882, only 45 (6.3) died. After operations only seven persons (0.9) died, and from accidental wound diseases there died 1.42 per cent.

THE only safety from the danger of trichinosis lies in having your meat thoroughly cooked. No curing or salting, no process of preservation will secure the eater of pork; cooking always will. Recently a French physician gave notice that he would eat any specimen of meat sent him, no matter if it were swarming with trichinæ. He did eat some very disgusting messes and without injury to himself. His safeguard was thorough cooking and his experiment ought to be thoroughly satisfactory.

